# *Faecalibacterium prausnitzii* Improves Lipid Metabolism Disorder and Insulin Resistance in Type 2 Diabetic Mice

**DOI:** 10.3389/bjbs.2023.10794

**Published:** 2023-03-21

**Authors:** Wenting Xuan, Yijing Ou, Wenting Chen, Lishan Huang, Chuangyu Wen, Guangying Huang, Wenting Tang, Daidi Zeng, Suran Huang, Lijuan Xiao, Zhongjun Li

**Affiliations:** ^1^ Department of Endocrinology, Affiliated Dongguan Hospital, Southern Medical University, Dongguan, China; ^2^ Department of Obstetrics and Gynecology, Affiliated Dongguan Hospital, Southern Medical University, Dongguan, China; ^3^ Dongguan Key Laboratory of Major Diseases in Obstetrics and Gynecology, Dongguan, China; ^4^ Guangdong Medical University, Zhanjiang, China

**Keywords:** inflammation, hepatic steatosis, *Faecalibacterium prausnitzii*, type 2 diabetic, insulin resistance, lipid metabolism

## Abstract

**Purpose:** Additional effective therapeutic strategies for Type 2 diabetes (T2D) patients are urgently needed*.* Gut microbiota plays an important role in T2D development and is a promising treatment strategy for T2D patients. Faecalibacterium prausnitzii (*F. prausnitzii*) is regarded as one of the most important bacterial indicators for a healthy gut, but the mechanisms of its anti-diabetic properties are still unclear.

**Methods and Results:** The abundance of *F. prausnitzii* in feces of patients with T2D was detected by using qPCR. The effects of *F. prausnitzii* on glucose homeostasis, insulin resistance (IR), dyslipidemia, hepatic steatosis and inflammation were investigated in type 2 diabetic (T2D) db/db mice. We also investigated *F. prausnitzii* in people. Our results showed that the abundance of *F. prausnitzii* was significantly lower in T2D patients compared to healthy subjects. In T2D mice, we found that *F. prausnitzii* treatment significantly decreased fasting blood glucose and IR index, indicating improved glucose intolerance as well as IR. Furthermore, based on evaluation of lipid-regulating enzyme activities and proinflammatory cytokine levels, *F. prausnitzii* was not only able to improve inflammation in both adipose tissue and liver, but also ameliorate hepatic steatosis through inhibiting the activity of hepatic lipogenic enzymes.

**Conclusion:** These results suggested that *F. prausnitzii* might serve as a therapeutic option for T2D by improved IR, lipid metabolism and inflammation.

## Introduction

Diabetes mellitus, a complex metabolic syndrome, has become a crucial public health concern worldwide due to the improvement of living standards and increasing aging population (1). The incidence of diabetes mellitus is increasing at a rapid rate with an estimated 700 million diabetic patients by 2045 (2). Type 2 diabetes (T2D) accounts for >90% of the total number of diabetic patients, which is predominantly induced by two main factors: insulin resistance (IR) and abnormal insulin secretion by pancreatic β-cells (3). IR is defined as the inability of insulin to regulate glucose homeostasis of target tissues, which is a systemic condition and asymptomatic resulting in extreme difficulties for prevention and treatment (4, 5). For the last few decades, only a few advanced developments have been made for the treatment of IR and it’s therefore necessary to find more effective therapeutic strategies for T2D patients (6–8).

Obesity is the most important risk factor for T2D, characterized by chronic low-grade inflammation leading to the secretion of proinflammatory factors involved in IR (9–11). T2D also increases the risk of cerebrovascular disease (12), cardiovascular disease (13) and non-alcoholic steatohepatitis (NASH) as well as non-alcoholic fatty liver disease (NAFLD) (14). Up to 70% of patients with T2D might present with NAFLD, which is highly correlated with the pathogenesis of IR, and around 20% of patients with T2D have non-alcoholic steatohepatitis (15, 16, 1). Due to the complexity of T2D and the inadequate understanding of the pathogenic mechanisms, there are limitations on providing therapeutic disease modifying options for T2D patients with NAFLD.

Gut microbiota has emerged as an important pathophysiological factor in T2D development, which is dominated by six main phyla including Bacteroidetes, Firmicutes, Actinobacteria, Fusobacteria, Verrucomicrobia and Proteobacteria (17, 18). *Faecalibacterium prausnitzii* (*F. prausnitzii*) is a crucial butyrate-producer of the phyla Firmicutes and the major member of the *Clostridium leptum* subgroup accounting for more than 5% of the total gut microbiota. It also plays an important role in the source of energy for the colonocytes and is considered as one of the most vital bacterial indicators for a healthy gut (19, 20). It’s reported that *F. prausnitzii* could secrete anti-inflammatory compounds (salicylic acid) to the surrounding environment and a decreased abundance of *F. prausnitzii* has been found to be associated with several human diseases, including celiac disease (19), inflammatory bowel disease (21), obesity (22) and diabetes (23). Interestingly, transplantation of *F. prausnitzii* has been applied as an intervention strategy for the treatment of dysbiosis of the gut’s microbial community, which precedes diabetes and autoimmune disease (24). In diabetic mice, a previous study demonstrated that *F. prausnitzii* could restore the gut barrier structure and function through the regulation of ZO-1 expression and tight junction pathway (25). However, the mechanism of anti-diabetic properties of *F. prausnitzii* is still unclear.

The aim of this study was to determine the effect of *F. prausnitzii* treatment on T2D-related metabolic changes in db/db mice and investigate whether the effects are associated with the regulation of metabolism of lipid and inflammation. Leptin receptor deficient mice (db/db mice) were used to develop a phenotype that is similar to T2D with symptoms including insulin resistance, obesity, hyperphagia, hyperinsulinemia and NAFLD, which were used in this study for evaluating the effect of *F. prausnitzii* treatment in the condition of T2D.

## Materials and Methods

### Patients and Specimen Collection

Fecal samples from 45 patients with T2D and 54 non-diabetic controls (all age and sex matched, 40–62 years old) from Affiliated Dongguan Hospital, Southern Medical University were collected. See Table 1 for detailed patient information. All 45 T2D patients were diagnosed using the World Health Organization diagnostic criteria for diabetes. The plasma glucose concentration of all 54 non-diabetic controls was evaluated by a fasting oral glucose tolerance test (OGTT). The exclusion criteria for our study were as follows: 1) history of inflammatory bowel diseases, 2) persistent diarrhea, or 3) use of antibiotics, or use of probiotic or prebiotic supplements within 3 months before the data collection. A 2-ml fecal sample from each participant was collected and stored at −80°C until use. Ethics approval was granted by the ethics committee of Affiliated Dongguan Hospital, Southern Medical University (KYKT2019-048). Written informed consent has been obtained from each subject. All experiments conform to the Declaration of Helsinki.

**TABLE 1:** Patient characteristics.

Characteristic	T2D patients (*n* = 45)	Non-diabetic controls (*n* = 54)	*P*
Age, y	50.2 (42–64)	48.7 (40–62)	NS
Sex, M/F	25/20	30/24	NS
BMI^a^	23.2 (17.98–29.64)	22.3 (16.13–28.59)-	NS
Diabetes duration, y	4.2 (1–27)		
Systolic blood pressure, mm Hg	115.3 (90–165)	113.5 (88–164)	NS
Diastolic blood pressure, mm Hg	74.4 (56–108)	73.7 (55–110)	NS
Random glucose level, mmol/L	15.30 (7.72–31.58)	5.46 (4.16–10.10)	<0.0001
Glycated hemoglobin level, %	11.31 (7.63–14.49)	5.62 (4.31–6.74)	0.001
Total cholesterol level, mmol/L	5.47 (4.35–6.73)	4.52 (3.34–6.22)	0.002
Serum triglyceride level, mmol/L	2.23 (0.93–7.30)	1.08 (0.62–4.84)	<0.0001
High-density lipoprotein cholesterol level, mmol/L	1.04 (0.73–1.50)	1.26 (0.62–2.20)	0.007
Low-density lipoprotein cholesterol level, mmol/L	3.05 (1.17–4.64)	2.60 (1.55–3.32)	0.035

^a^ Body mass index (BMI) is calculated as the weight in kilograms divided by the square of the height in meters.

Data are no. of subjects or median (range).

Abbreviation: NS, not significant.

All participants completed an interview to determine health status, lifestyle, and medication use. All patients with diabetes received diabetes education and followed a diabetes diet. Dietary intake patterns were determined using a food frequency questionnaire.

### Fecal DNA Extraction and Real-Time Quantitative PCR (qPCR)

Total fecal DNA extraction was performed using the DNA Stool Mini Kit according to the manufacturer’s instructions (Tiangen, Beijing, China). qPCR was performed on a LightCycler^®^480 II (Roche Applied Science, Indianapolis, IN, United States) using a SYBR green-based assay (Bio-Rad, Hercules, CA, United States). Measurements were performed in triplicates for each sample. Primers were as follows: *F. prausnitzii*, forward: 5′-ATC​GCC​CTG​TGG​ATG​ACT​GA-3'; reverse: 5′-GCC​AGG​AGA​AAT​CAA​ACA​G-3'. Plasmid DNA containing the respective amplicon was diluted in 10-fold increments (108-101 copies) and used as quantification standards. Universal 16S rDNA was used as internal control and the abundances of gene biomarkers were expressed as relative levels to 16S rDNA.

### *In Vitro* Culture

*F. prausnitzii* (ATCC 27766) was inoculated in a basal mucin-based medium and incubated under anaerobic conditions. Anaerobic PBS with 25% (vol/vol) glycerol under strict anaerobic conditions was used to wash and concentrate the cultures. An identical quantity of *F. prausnitzii* was grown on the synthetic medium and was inactivated by pasteurization for 30 min at 70°C. And then cultures were immediately frozen and stored at −80°C. We thawed glycerol stocks under anaerobic conditions and diluted them with anaerobic PBS to an end concentration of 1 × 10^8^ CFU/150 μL and 2.5% glycerol before they were administrated by oral gavage.

### Animals

Eighteen male 4-week-old C57BL/KsJ-db/db mice and C57BL/6 mice were purchased from Model Animal Center of Nanjing University (Nanjing, Jiangsu, China). The mice were randomly divided into 3 groups (*n* = 6) and fed the respective experimental diets for 5 weeks: C57BL/6 mice were used as a non-diabetic control (ND), C57BL/KsJ-db/db mice supplemented with PBS (T2D) and C57BL/KsJ-db/db mice supplemented with F. prausnitzii (FP, 108 CFU/150 μL; T2D-FP) by oral gavage three times each week. They were allowed free access to food and tap water. Food intake and bodyweight were measured daily and weekly, respectively. All mice were anesthetized with isoflurane (5 mg/kg body weight) after a 12-h fast at the end of experimental period and blood samples were collected from the inferior vena cava for the quantification of blood and plasma biomarkers. Adipose tissue (retroperitoneal, perirenal, subcutaneous, mesentery, and scapular white adipose tissue) were promptly removed, weighed and kept at −80°C. The livers were also removed and fixed for immunohistochemistry after 5 weeks of treatment. The ethics committee of Guangdong Medical University specifically approved this study.

### Blood and Plasma Parameters

Blood was drawn from the tail vein and the concentration of blood glucose was monitored using a glucometer (Roche Diagnostics, Brussels, Belgium) every week after a 12-h fast. Intraperitoneal glucose tolerance test was performed on week 4. After a 12-h fast, mice were injected intraperitoneally with glucose (0.5 g/kg body weight). Blood glucose levels were determined at 0, 20, 40, and 60 min after glucose injection. At the end of the experimental period, the homeostatic model assessment index of IR (HOMA-IR) was calculated as follows: HOMA-IR = [fasting glucose (mmol/L) × fasting insulin (µL U/mL)]/22.51 (26). The levels of cytokines (tumor necrosis factor (TNF)-α, Interleukin (IL)-6, IL-12p70, and interferon (IFN)-γ) in the plasma were determined by using a multiplex detection kit from Bio-Rad Laboratories (Hercules, CA, United States) and analyzed with a Luminex 200 Lab map system (Luminex, Austin, TX, United States).

### Plasma and Hepatic Lipids

The levels of free fatty acids were detected by Wako enzymatic kit (Seebio, Shanghai, China). The levels of total cholesterol, triglyceride, and high-density lipoprotein (HDL)-cholesterol were determined by an enzymatic method (Sigma, St. Louis, MO, United States). The atherogenic index was calculated as follows: atherogenic index = [(total cholesterol)-(HDL-cholesterol)]/(HDL-cholesterol) (27). Hepatic lipids were extracted and analyzed using the same enzymatic kits for plasma analyses.

### Hepatic Enzymatic Activity

Fatty acid synthase activity was evaluated by monitoring the malonyl-CoA-dependent oxidation of NADPH at 340 nm (nmol/min/mg protein). Malic enzyme activity was determined by mixing the cytosolic enzyme mixed with 0.2 mM triethanolamine buffer (pH 7.4),1.5 mM L-malate, 12 mM MnCl_2_, and 680 M NADP^+^, and analyzing for 1 min at 340 nm (26°C) on a spectrophotometer. The activity of carnitine palmitoyltransferase was expressed as nmol/min/mg protein. The activity of β-oxidation activity was evaluated spectrophotometrically by monitoring the reduction of NAD to NADH in the presence of palmitoyl-CoA.

### RNA Extraction and Analysis of Gene Expression

The total RNA in liver and epididymal adipose tissues were extracted using Trizol Reagent (Invitrogen, Carlsbad, CA, United States) and quantified with a Nanodrop spectrophotometer (NanoDrop 2000, Thermo Scientific, Waltham, MA, United States). The RNA was reverse-transcribed into complementary DNA using PrimeScript™ RT Master Mix (TaKaRa, Kusatsu, Shiga, Japan). Expression of mRNA was measured by using TB Green™ Kit (TaKaRa). The quantification of the target and reference (GAPDH) genes was performed in triplicate using a LightCycler^®^ 480 II (Roche Diagnostics). Primers used were as follows: GAPDH, forward, 5′- CTG​GGC​TAC​ACT​GAG​CAC​C-3′, reverse, 5′-GGA​ACG​CTT​CAC​GAA​TTT​G-3; IL6, forward, 5′- AAG​TGG​TCG​TTG​AGG​GCA​ATG-3′, reverse, 5′- GCT​TGG​CGT​GTG​GTC​TTT-3’; TNFα, forward, 5′- GGT​GCC​TAT​GTC​TCA​GCC​TCT​T-3′, reverse, 5′- GCC​ATA​GAA​CTG​ATG​AGA​GGG​AG-3’. All the primers were synthesized by Shanghai Shenggong Bioengineering Co., Ltd. 2^−ΔΔCt^ method was utilized for data assessment.

### Morphological Analysis of the Liver

After 5 weeks of treatment, the livers were removed from the mice, fixed in 10% formalin and embedded in paraffin, which were processed routinely for paraffin embedding. 4-µm sections were prepared and dyed with hematoxylin-eosin (H&E). Stained areas were viewed using an optical microscope with a magnifying power of ×200 and ×400.

### Statistical Analysis

All data were displayed as mean ± standard deviation (SD) from at least three independent experiments in triplicate. Data were analyzed by paired t test between two groups and by one-way analysis of variance (ANOVA) followed by Bonferroni test for multiple comparison. Statistical analyses were performed with SPSS 22.0 (IBM, Armonk, NY, United States) for Windows. *p* < 0.05 was considered statistically significant.

## Results

### Abundance of *F. prausnitzii* in Patients With T2D

**FIGURE 1:**
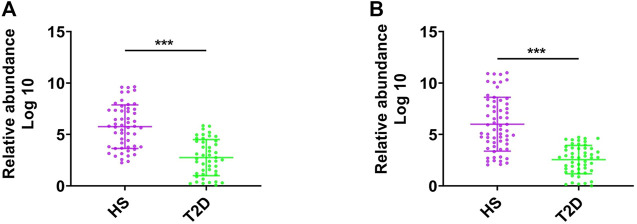
The abundance of *F. prausnitzii* in feces of patients with type-2 diabetes (T2D). **(A)** The abundance of *F. prausnitzii* in feces of patients with type-2 diabetes (T2D) and healthy subjects (HS). **(B)** The abundance of *F. prausnitzii* in GMrepo T2D cohort. Error bars, SD. ***p < 0.001.

We first assessed the abundance of *F. prausnitzii* in feces of patients with T2D by a qPCR assay in two independent patient cohorts. As shown in Figure 1A, the abundance of *F. prausnitzii* was significantly lower in T2D patients (*n* = 45) compared to healthy subjects (HS, *n* = 52, *p* < 0.001). GMrepo (data repository for Gut Microbiota) is a database of curated and consistently annotated human gut metagenomes. Consistent with our findings, the abundance of *F. prausnitzii* was also found to be significantly reduced in GMrepo data set of T2D patient samples (*n* = 50) and healthy subject samples (*n* = 62) (Figure 1B). These results suggested that the abundance of *F. prausnitzii* was significantly decreased in T2D patients and may be implicated in the progression of T2D.

### Effect of *F. prausnitzii* Supplementation on Glycemic Homeostasis and IR

**FIGURE 2:**
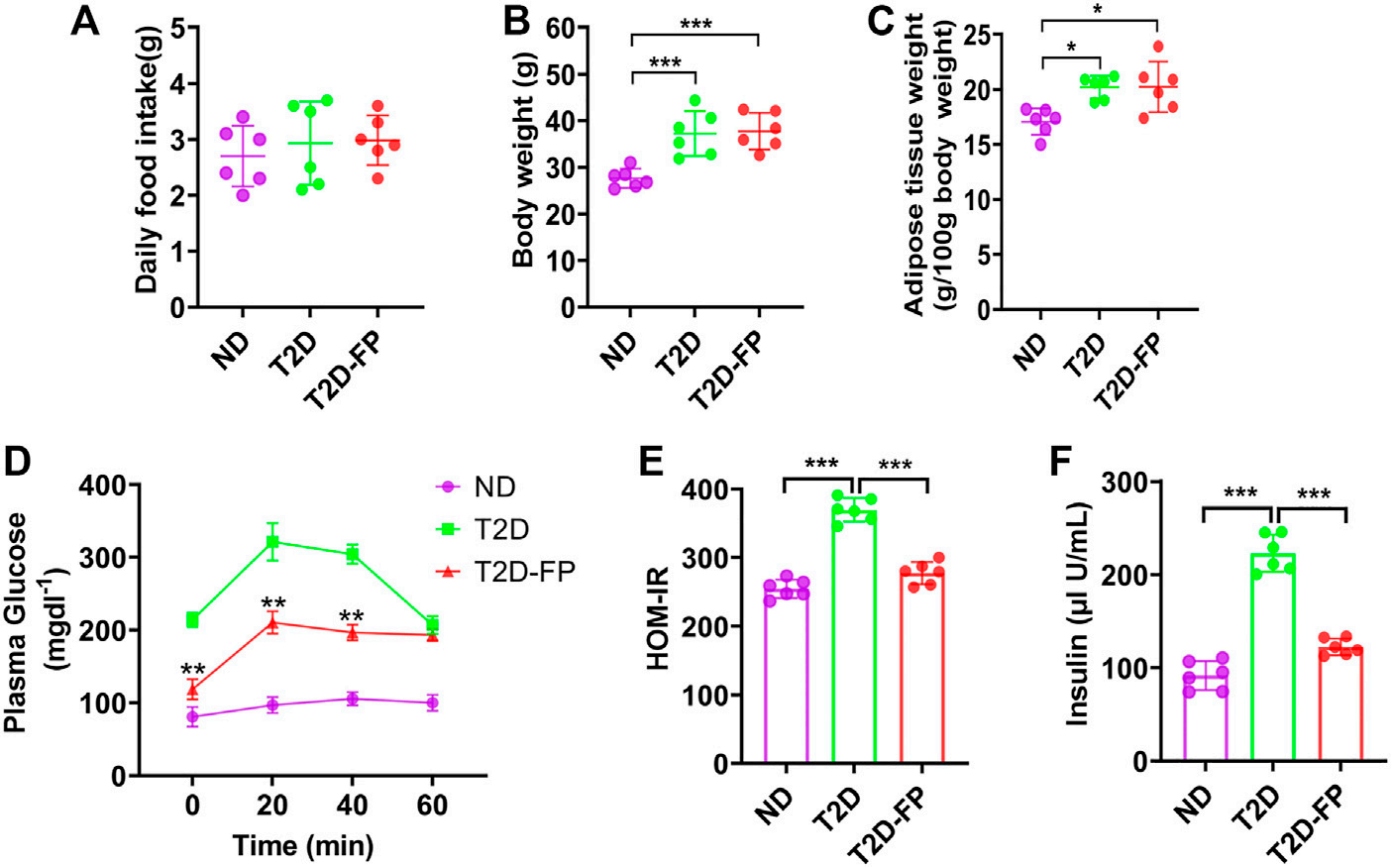
*F. prausnitzii* restored insulin resistance of T2D mice. Effect of dietary *F. prausnitzii* on daily food intake **(A)**, body weight **(B)**, adipose tissue weight **(C)**, blood glucose by IPGTT **(D)**, **p < 0.01, T2D-FP v.s. T2D, HOMA-IR **(E)** and plasma insulin levels **(F)** in ND, T2D and T2D-FP mice. Error bars, SD. *p < 0.05, ***p < 0.001.

Based on the lower abundance of *F. prausnitzii* in T2D patients compared with HS, we hypothesized that *F. prausnitzii* may play a positive role in T2D. Herein, we further explored the effect of *F. prausnitzii* on T2D. *F. prausnitzii* supplementation was given to db/db mice (T2D-FP mice). T2D-FP mice showed similar food intake, as compared to both ND and T2D mice (Figure 2A). However, T2D and T2D-FP mice showed significantly increased body weight and adipose tissue mass compared to ND (Figures 2B,C). From the results of the intraperitoneal glucose tolerance test, T2D-FP group showed significantly lower glucose intolerance compared to the T2D mice (Figure 2D). *F. prausnitzii* could decrease IR compared to the T2D mice by using HOMA-IR method, indicating a lower IR index in response to *F. prausnitzii* c (Figure 2E). *F. prausnitzii* also decreased the plasma insulin levels compared to the T2D mice (Figure 2F). These results demonstrated that *F. prausnitzii* restored glycemic homeostasis in T2D without attenuation of body weight.

### Effects of *F. prausnitzii* on Dyslipidemia and Hepatic Steatosis

**FIGURE 3:**
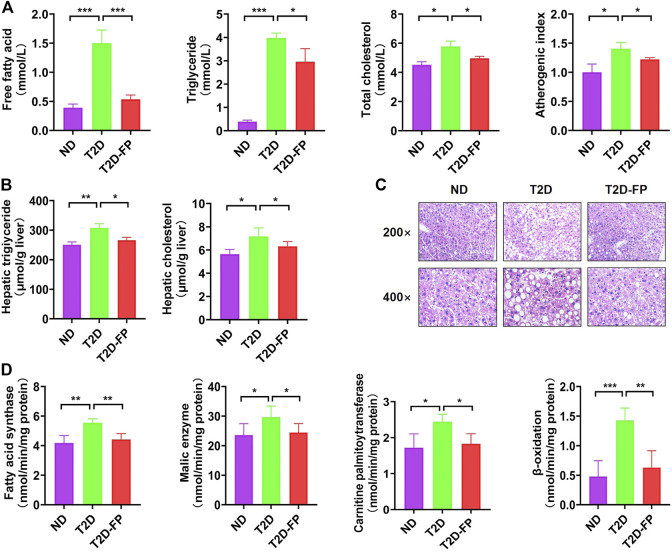
*F. prausnitzi* modulated dyslipidemia and hepatic steatosis of T2D mice. Effect of dietary *F. prausnitzii* on plasma lipid levels **(A)**, hepatic lipid content **(B)**, hepatic morphology **(C)**, and hepatic lipid-regulating enzyme activity **(D)** in ND and T2D mice. Error bars, SD. *p < 0.05, **p < 0.01, ***p < 0.001.

As shown in Figure 3A, T2D mice showed significantly higher levels of plasma free fatty acid, triglyceride, total cholesterol and increased atherogenic index, as compared to ND mice. In contrast, *F. prausnitzii* treatment significantly lowered the levels of plasma free fatty acid, triglyceride levels, total cholesterol and decreased atherogenic index compared to T2D mice. *F. prausnitzii* treatment showed a significant decrease in both hepatic triglyceride and cholesterol content compared to T2D mice (Figure 3B). Obvious lipid droplet accumulation was observed in the liver of T2D mice by morphological analysis, while treatment with *F. prausnitzii* exhibited a significant decrease in lipid droplet accumulation compared to T2D mice (Figure 3C).

We also evaluated the activity of lipid-regulating enzymes and fatty acid oxidation in the liver with the aim of clarifying the mechanism of the decreased hepatic lipid accumulation by *F. prausnitzii*. Significant increases were observed in both lipogenic fatty acid synthase, malic enzyme activity, carnitine palmitoyltransferase and β-oxidation in the livers of T2D mice, whereas treatment with *F. prausnitzii* showed a significant decrease in all of them, compared to T2D mice (Figure 3D). These findings indicated that *F. prausnitzii* supplementation improved fatty acid metabolism disorder in T2D mice.

### Effect of *F. prausnitzii* Supplementation on Inflammation

**FIGURE 4:**
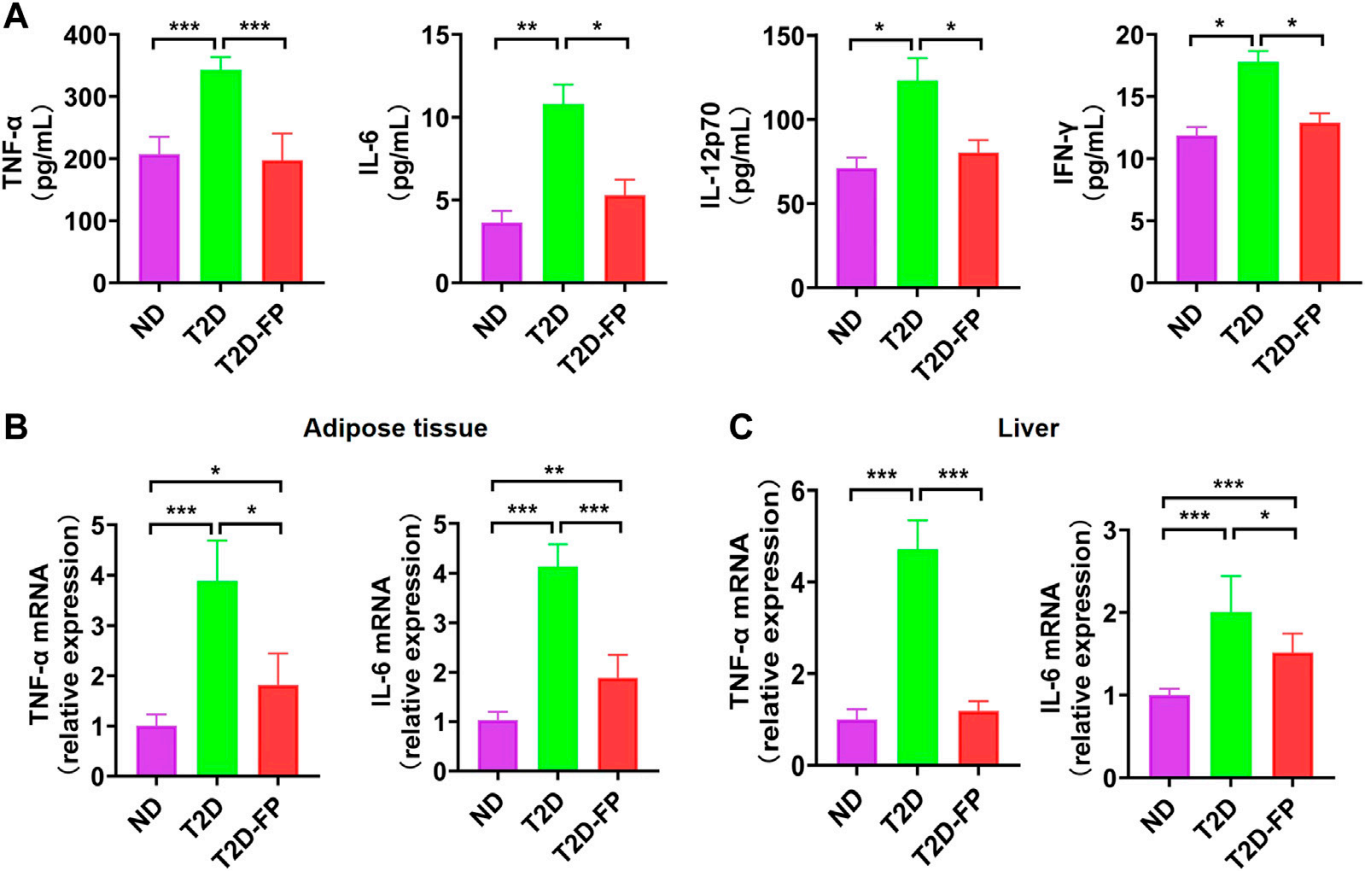
*F. prausnitzii* attenuated pro-inflammatory cytokine expression in T2D mice. Effect of dietary *F. prausnitzii* on plasma pro-inflammatory cytokine levels **(A)**, and the mRNA expression of pro-inflammatory cytokines in adipose tissue and livers **(B,C)** of ND and T2D mice. Error bars, SD. *p < 0.05, **p < 0.01, ***p < 0.001.

In previous studies, low abundance of *F. prausnitzii* could support inflammatory processes which potentially results in gastrointestinal tract disorders, such as inflammatory bowel disease, irritable bowel syndrome as well as colorectal cancer (28–30). *F. prausnitzii* has been reported to be a crucial butyrate-producer with anti-inflammatory effect (31). In this study, the levels of pro-inflammatory cytokines such as TNF-α, IL-6, IL12p70, and IFN-γ were significantly decreased in the plasma of the T2D-FP mice compared to the T2D mice (Figure 4A). Moreover, T2D-FP mice significantly down-regulated the mRNA expression of TNF-α and IL-6 in adipose tissue and the liver compared to the T2D mice (Figures 4B, C). These results demonstrated that *F. prausnitzii* supplementation relieved inflammation in T2D mice.

## Discussion

This study demonstrated that supplementing mouse diets with *F. prausnitzii* for 5 weeks could significantly decrease the levels of plasma glucose and insulin, improving the glucose intolerance and insulin resistance (IR) index compared to T2D mice-obesity-induced diabetic animals with IR. We also clarified that *F. prausnitzii* was able to ameliorate inflammation and improve hepatic steatosis *via* suppressing the activity of hepatic lipogenic enzymes.

Some clinical indicators like fasting blood glucose, 2 hours postprandial blood glucose and IR have been widely applied in the diagnosis of T2D. However, T2D may have developed for years when the abnormalities of those clinical indicators are detected. Considering the complexity of T2D, there is an urgent need to find some new promising biomarkers for T2D to provide a theoretical basis for the early diagnosis and risk assessment (32, 3). It has been proposed that *F. prausnitzii* may serve as a biomarker for assisting diagnostics in gut diseases (33). In this study, the abundance of *F. prausnitzii* was significantly decreased in T2D patients, which may implicate the progression of T2D and be a potential diagnostic biomarker for T2D.

IR means decreased responsiveness or sensitivity to the metabolic process of insulin including inhibition of hepatic glucose production and insulin-mediated glucose metabolism. Due to the effects of IR, IR has been reported to relate with T2D, obesity, coronary artery disease, hypertension and dyslipidemias (34). We showed that *F. prausnitzii*-treated mice had a lower glucose intolerance and IR index, which indicated that *F. prausnitzii* might restore the glycemic homeostasis without attenuation of body weight gain.

T2D is strongly associated with obesity, IR, metabolic syndrome and NAFLD(35). Hepatic cell dysfunction might directly induce insulin resistance thereby leading to T2D development (36). For example, impaired inhibition of hepatic glucose production is correlated with increased intrahepatic triglyceride accumulation, which is regarded as a hallmark of NAFLD. Intrahepatic triglyceride accumulation is also considered as an important indicator of insulin action in adipose tissue, liver and skeletal muscles (37). Triglyceride accumulation is the result of an imbalance between lipid removal (i.e., consumption of fatty acid by mitochondrial β-oxidation) and lipid acquisition (i.e., *de novo* lipogenesis and fatty acid uptake) (38). IR fails to inhibit gluconeogenesis but still continues to stimulate lipogenesis resulting in hyperinsulinermia and hyperglycemia (39). It’s reported that the db/db mice used in our study exhibited higher hepatic triglycerides and increased levels of lipogenic proteins and genes such as fatty acid synthase compared to db/+ mice (40, 41). Our results demonstrated that *F. prausnitzii* can significantly downregulate the hepatic lipid-regulating enzyme activity, including fatty acid synthase, malic enzyme, carnitine palmitoyltransferase and β-oxidation in the livers of db/db mice, indicating that the reduced hepatic triglyceride levels produced by *F. prausnitzii* are accounted for by the decrease in hepatic lipogenesis and responsible in improving hepatic steatosis.

It has been shown that T2D is a low-grade chronic inflammation state and the inflammatory factors, including IL, TNF, adiponectin and resistin, can be detected in T2D. These inflammatory factors are able to activate intracellular/threonine kinases and catalyze the phosphorylation of vital proteins of the insulin signaling pathway, leading to IR (3). Interestingly, a previous study has clarified that *F. prausnitzii* was correlated with inflammatory markers, which might play a role in low-grade inflammation pathologies such as diabetes and obesity (42). The anti-inflammatory effects of *F. prausnitzii* may be partly due to the secretion of proinflammatory mediators and the inhibition of nuclear factor-κB by the secreted metabolites. Supernatant of *F. prausnitzii* or oral administration of *F. prausnitzii* was able to increase the production of anti-inflammatory cytokine IL-10 and decrease the production of proinflammatory mediator, such as IL-12 and IFN-γ (43). In this study, the levels of TNF-α, IL-6, IL-12p70, and IFN-γ in plasma were downregulated by *F. prausnitzii* treatment. The mRNA expression levels of TNF-α and IL-6 in adipose tissue were also downregulated by *F. prausnitzii* treatment, indicating that decreasing these proinflammatory cytokines expression in adipose tissue may be responsible for the improvement of IR by *F. prausnitzii*. The mRNA expression levels of TNF-α and IL-6 in the liver were also downregulated by *F. prausnitzii* treatment, indicating that the hepatic inflammation was reduced.

## Conclusion

In conclusion, we have demonstrated a critical role for *F. prausnitzii* in improving IR and glucose intolerance in T2D. *F. prausnitzii* was not only able to improve inflammation in both adipose tissue and liver, but also ameliorate hepatic steatosis through inhibiting the activity of hepatic lipogenic enzymes, which might serve as a potential therapeutic strategy for T2D.

## Data Availability

All data generated or analysed during this study are included in this published article.
